# Prevalence of current smoking and associated factors in older adults in Brazil

**DOI:** 10.1590/1806-9282.20240372

**Published:** 2024-09-13

**Authors:** José Arthur Didoné Machado, João Vitor Fantin, Juliana Coelho de Campos, Eliane Traebert, Cesar de Oliveira, Jefferson Traebert

**Affiliations:** 1 Universidade do Sul de Santa Catarina, School of Medicine – Palhoça (SC), Brazil.; 2 Universidade do Sul de Santa Catarina, Graduate Program in Health Sciences– Palhoça (SC), Brazil.; 3 University College London, Department of Epidemiology and Public Health – London, United Kingdom.

**Keywords:** Smoking, Older adults, Chronic disease, Aging, Tobacco

## Abstract

**OBJECTIVE:**

The aim of this study was to estimate the prevalence of current smoking and its associated factors in adults aged 50 years and older in Brazil.

**METHODS:**

This cross-sectional study utilized data from the ELSI-Brazil study, encompassing 9,412 adults aged 50 years or over. A multivariate model using Poisson regression with a robust estimator was employed, estimating prevalence ratios and their 95% confidence intervals.

**RESULTS:**

The prevalence of current smoking was 17.04%. It was positively and independently associated with male gender, age up to 62 years, living without a partner, illiteracy, chronic obstructive pulmonary disease, depression, poor or very poor sleep quality, and alcohol intake more than once a month. Conversely, systemic arterial hypertension, hypercholesterolemia, diabetes mellitus, and repetitive strain injuries showed an inverse and independent association with current smoking.

**CONCLUSION:**

The prevalence of current smoking among adults over 50 years old in Brazil was approximately 17%, with associations found with certain sociodemographic conditions and self-reported comorbidities.

## INTRODUCTION

Older adults are more vulnerable to chronic degenerative diseases^1^, and smoking is a risk factor closely associated with these diseases^2,3^. Worldwide, 1.14 billion people were tobacco consumers in 2019 associated with 7.69 million deaths and approximately 200 million cases of working disability^4^. Tobacco effects account for more than 300,000 deaths and 2.2 million illnesses per year in Latin America resulting in economic damage^5^.

Brazil also experienced a drop in the rate of adult smokers^6^. From 1986 onward, several tobacco control initiatives were implemented in Brazil causing a decrease in the rate from 34.8% in 1989 to 18.2% in 2008^7^ and 12.8% in 2019^8^. Brazil was the second member of the World Health Organization that implemented successful laws to control tobacco use^9^.

Data from Vigitel (Brazilian Surveillance of Risk and Protective Factors for Chronic Diseases by Telephone Survey) indicated reductions across all age groups and educational levels. The most significant decreases were observed among adults aged 45–54 years, declining from 22.8% in 2006 to 9.1% in 2023^10^.

In contrast, the Brazilian Longitudinal Study of Aging (ELSI-Brazil) aimed to examine the aging process of the Brazilian population from both social and biological perspectives and its implications for adults aged 50 years or older. The objective of this study was to estimate the prevalence of current smoking and identify associated risk factors among individuals aged 50 years and older in Brazil.

## METHODS

This is a cross-sectional study nested within the Brazilian Longitudinal Study of Aging (ELSI-Brazil)^11^. Our investigation used ELSI's baseline data that was collected in 2015–2016 by the Oswaldo Cruz Foundation and the Federal University of Minas Gerais. Data were collected on individuals aged 50 years or older residing in 70 municipalities across 21 states and the Federal District. The sample was designed to be representative of community-dwelling Brazilians aged 50 years or older. To ensure representation across urban and rural areas of municipalities of various sizes, the ELSI-Brazil sampling employed a multistage design. This design involved the stratification of primary sampling units (municipalities), census tracts, and households. Municipalities were divided into four strata based on population size: the first stratum (≤26,700 inhabitants from 4,420 municipalities), the second stratum (26,701–135,000 inhabitants from 951 municipalities), the third stratum (135,001–750,000 inhabitants from 171 municipalities), and the fourth stratum (>750,000 inhabitants from 23 municipalities). All residents aged 50 years or older in the selected households were eligible for the interview. An inverse sampling design was employed to mitigate nonresponse bias without enlarging the sample size. The final sample consisted of participants from 70 municipalities across the major regions of Brazil. Sample weights were derived to account for differential probabilities of selection and nonresponse. For further methodological details, including sampling procedures, refer to Lima-Costa et al^12^. The total study population data referred to 9,412 of both genders, who were included in the ELSI-Brazil database. Authorization to use the data was obtained through access registration on the study's official website: http://elsi.cpqrr.fiocruz.br/instrucoes-para-uso/.

Data collection was performed using an individual questionnaire and a household questionnaire that can be seen at https://elsi.cpqrr.fiocruz.br/questionario/. The dependent variable in our study was smoking (as reported: yes/no) according to the following question, "*Do you currently smoke?*" in the following context: "*To finish this section, I will ask you some questions about smoking industrialized cigarettes, straw cigarettes or other tobacco products that are smoked, such as cigars, cigarillos, pipes, clove (or Bali) cigarettes, Indian cigarettes (or bidis) and hookah (or water pipes). Please do not respond about smokeless tobacco products such as snuff and chewing tobacco. Do not consider electronic cigarettes*."

The independent variables were age (categorized in the median of the distribution, which was 62 years of age), skin color (categorized as white/non-white), marital status (with or without a partner), education (categorized in up to 8 years and more than 8 full years of study), remunerated work (yes/no), self-reported comorbidities in response to questions formulated as follows for each comorbidity included: "*Has a doctor ever told you that you have arterial hypertension (high blood pressure)?*" or "*Has a doctor ever told you that you have diabetes (blood sugar)?*" (has/does not have systemic arterial hypertension, hypercholesterolemia, asthma, chronic obstructive pulmonary disease, acute myocardial infarction, cardiac insufficiency, stroke, cancer, diabetes mellitus, depression, Alzheimer's disease, Parkinson's disease, repetitive strain injury), sleep quality (regular/good/very good or bad/very bad), and alcohol intake (no, once a month, or more than once a month).

**Figure 1:**
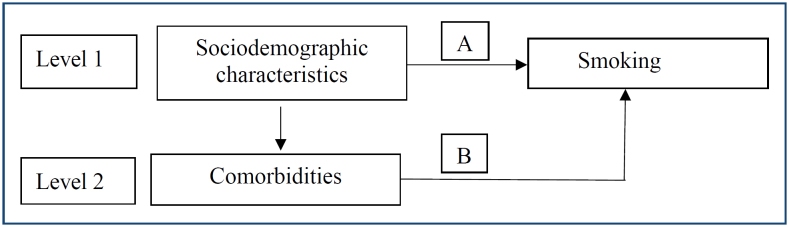
Proposal for a hierarchical analysis of smoking prevalence and associated factors in older adults aged 50 years and over.

Data were analyzed in the software SPSS Statistics for Windows, 18.0 (SPSS Inc., Chicago, IL, USA). Bivariate analyses between the dependent and independent variables were performed using the chi-square test to observe proportional homogeneity. All variables that were statistically significant with p<0.05, as well as those with p<0.20, were included in a multivariate model using Poisson regression with a robust estimator. These variables were ranked according to the stepwise forward adjustment technique, as proposed for hierarchical analysis of smoking prevalence and associated factors (Figure 1).

ELSI-Brazil was approved by the Research Ethics Committee of the *Centro de Pesquisas René Rachou da Fundação Oswaldo Cruz* (protocol 34649814.3.0000.5091).

## RESULTS

Data from 9,412 individuals aged 50 years or older from the ELSI-Brazil database was included. The average age was 63.5 years (SD=10.1), the median was 62 years, and 56.5% were female. The average level of education was 7 completed years of study (SD=8.7) and 16.4% were illiterate. The reported current smoking prevalence was 17.04% (95%CI 16.30–17.70) or 1,604 participants. Table 1 presents the bivariate analysis between sociodemographic variables, comorbidities, and smoking.

**Table 1:** Sociodemographic variables, comorbidities, and smoking (Brazilian adults aged 50 years and over).

Variables		Smoking				
		n	%	PR_c_ *	95%CI**	p-value
First level—sociodemographic						
Gender						
	Male	843	20.6	1.04	1.03–1.05	<0.001
	Female	761	14.4	1.00		
Skin color						
	White	564	15.7	1.00	1.01–1.03	0.012
	Not white	972	17.7	1.02		
Age (median)						
	Over 62 years	566	12.7	1.00	1.04–1.06	<0.001
	Up to 62 years	1.038	21.0	1.05		
Education						
	More than 8 years schooling completed	341	14.8	1.00	1.01–1.03	0.300
	From 1 to 8 years schooling completed	965	17.5	1.02		0.042
	Illiterate	284	18.6	1.03	1.01–1.04	<0.001
Marital status						
	With partner	845	15.5	1.00	1.01–1.03	<0.001
	No partner	759	19.1	1.02		
Remunerated activity						
	No	1.043	15.8	1.00	1.01–1.03	<0.001
	Yes	561	19.9	1.02		
Second level—comorbidities						
Systemic arterial hypertension						
	No	910	20.8	1.00	0.95–0.97	<0.001
	Yes	687	13.7	0.96		
Hypercholesterolemia						
	No	1.182	18.4	1.00	0.96–0.98	<0.001
	Yes	403	13.9	0.97		
Asthma						
	No	1.514	17.0	1.00	0.98–1.02	0.587
	Yes	85	17.9	1.01		
Chronic obstructive pulmonary disease						
	No	1.478	16.7	1.00	1.01–1.04	0.001
	Yes	121	22.2	1.03		
Acute myocardial infarction						
	No	1.502	17.0	1.00	0.98–1.02	0.549
	Yes	100	18.0	1.01		
Cardiac insufficiency						
	No	1.508	17.4	1.00	0.97–1.02	0.005
	Yes	89	13.1	0.98		
Stroke						
	No	1.521	17.2	1.00	0.98–1.01	0.275
	Yes	82	15.3	0.99		
Cancer						
	No	1.534	17.3	1.00	0.95–0.97	0.008
	Yes	64	12.7	0.97		
Diabetes mellitus						
	No	1.391	17.8	1.00	0.96–0.99	<0.001
	Yes	201	13.2	0.97		
Depression						
	No	1.263	16.5	1.00	1.01–1.03	0.002
	Yes	335	19.6	1.02		
Alzheimer's disease						
	No	1.593	17.1	1.00	0.92–0.99	0.040
	Yes	7	8.5	0.95		
Parkinson's disease						
	No	1.588	17.0	1.00	0.96–1.06	0.715
	Yes	12	18.8	1.01		
Repetitive strain injury						
	No	487	19.9	1.00	0.97–0.99	<0.001
	Yes	1.075	16.3	0.98		
Sleep quality						
	Regular/good/very good	1.246	16.3	1.00	1.01–1.03	<0.001
	Bad/very bad	355	20.2	1.02		
Alcohol intake						
	No	939	13.6	1.00		
	Once a month	127	23.3	1.03	1.01–1.05	0.032
	More than once a month	535	27.4	1.08	1.07–1.09	<0.001

* PR_c_: Crude prevalence ratio.

** 95%CI: 95% confidence interval.

Table 2 displays the results of the multivariate analysis between sociodemographic variables, comorbidities, and current smoking in the final hierarchical model. The variables that were positively, statistically, and independently associated with a higher prevalence of current smoking were male gender (PR=1.03; 95%CI 1.02–1.04) (p<0.001), age up to 62 years of age (PR=1.04; 95%CI 1.03–1.05) (p<0.001), living without a partner (PR=1.04; 95%CI 1.03–1.05) (p<0.001), illiteracy (PR=1.04; 95%CI 1.02–1.06) (p<0.001), presence of chronic obstructive pulmonary disease (PR=1.03; 95%CI 1.01; 1.04) (p=0.011), depression (PR=1.03; 95%CI 1.01; 1.04) (p<0.001), bad or very bad sleep quality (PR=1.02; 95%CI 1.01–1.03) (p=0.001), and alcohol intake more than once a month (PR=1.08; 95%CI 1.05–1.09) (p<0.001). Variables associated with lower prevalence of current smoking in a statistically significant and independent way were systemic arterial hypertension (PR=0.97; 95%CI 0.96; 0.98) (p<0.001), hypercholesterolemia (PR=0.98; 95%CI 0.97–0.99) (p<0.001), diabetes mellitus (PR=0.98; 95%CI 0.97–0.99) (p=0.040), and repetitive strain injury (PR=0.98; 95%CI 0.96–0.99) (p=0.004).

**Table 2:** Sociodemographic variables, comorbidities, and current smoking in Brazilian older adults aged 50 years and over (Final model).

Variables		Current smoking		
		PR_a_ *	95%CI**	p-value
First level—sociodemographic				
Gender				
	Female	1.00	1.02–1.04	<0.001
	Male	1.03		
Age (median)				
	Over 62 years old	1.00	1.03–1.05	<0.001
	Up to 62 years	1.04		
Marital status				
	With partner	1.00	1.03–1.05	<0.001
	No partner	1.04		
Education				
	More than 8 schooling completed	1.00		
	From 1 to 8 schooling completed	1.01	0.99–1.03	0.081
	Illiterate	1.04	1.02–1.06	<0.001
Second level—self-reported comorbidities				
Systemic arterial hypertension				
	No	1.00	0.96–0.98	<0.001
	Yes	0.97		
Hypercholesterolemia				
	No	1.00	0.97–0.99	<0.001
	Yes	0.98		
Chronic obstructive pulmonary disease				
	No	1.00	1.01–1.05	0.011
	Yes	1.03		
Cancer				
	No	1.00	0.97–1.00	0.061
	Yes	0.98		
Diabetes mellitus				
	No	1.00	0.97–0.99	0.040
	Yes	0.98		
Depression				
	No	1.00	1.01–1.04	<0.001
	Yes	1.03		
Repetitive strain injury				
	No	1.00	0.96–0.99	0.004
	Yes	0.98		
Sleep quality				
	Regular/good/very good	1.00	1.01–1.03	0.001
	Bad/very bad	1.02		
Alcohol intake				
	No	1.00		
	Once a month	1.02	0.99–1.04	0.071
	More than once a month	1.07	1.05–1.09	<0.001

* PR_a_: adjusted prevalence ratio.

** 95%CI: 95% confidence interval.

## DISCUSSION

The current smoking prevalence in Brazilian adults aged 50 years and older found in the present study was 17.04%. The PLATINO study (*Proyecto Latinoamericano de Investigación en Obstrucción Pulmonar*) in the city of São Paulo/SP in 2003 showed that around 24.0% of people aged 40 years or over were smokers, while 33.1% were ex-smokers^13^. In a recent study carried out in Japan, similar data were presented, indicating a prevalence of 13.8% of smoking among older adults^14^. The prevalence of current smoking in older adults in Brazil follows rates that are similar to those rates in other countries.

Regarding gender, a higher smoking prevalence was found among men, as shown in another Brazilian study^15^. This is in line with studies carried out with American older adults^15-17^. The epidemic history of tobacco shows that it has increased first among males and later among females^18^.

In our study, age was dichotomized in the median of the distribution with a value of 62 years, observing a higher prevalence of smoking in those individuals aged up to 62 years. A previous study carried out in Brazil presents similar data with a lower prevalence in older adults. In this same study, the results show that the lower the level of education, the greater the prevalence of smoking. The higher the level of education, the greater the access to information on the risks of tobacco use; perhaps this is the condition behind the lower smoking practice, as it is believed that it is through knowledge and education that people become aware and, therefore, start to abandon practices that increase the risk of diseases and adopt others that generate health.

This study demonstrated that relationships can positively influence healthy habits. In addition, another study presents results showing that marriage is a protective factor for quitting smoking, thus reducing smoking rates^19^.

In addition, a positive association was observed between smoking and alcohol consumption. A study in which the combined consumption of alcohol and tobacco was adopted as the dependent variable showed that the increased prevalence of both substances’ consumption converged in the bivariate analysis when correlated with other independent variables, such as education^20^. The association between both substances may be due to the social influence that smoking and alcohol have in common.

Regarding self-reported comorbidities, a higher prevalence of smoking was observed in individuals with some chronic diseases. However, it is important to highlight that self-reporting of chronic diseases is related to the possibility of underdiagnosis or even overdiagnosis. A study carried out in Canada showed that female smokers were more prone to the development of chronic diseases, chronic obstructive pulmonary disease, and lung cancer^19^. Male smokers are more likely to develop comorbidities, including chronic obstructive pulmonary disease and lung cancer^21^. A study conducted in the United States showed that smokers were more likely to develop depression^22^.

A few other diseases were associated with a reduced rate of smoking, such as hypercholesterolemia, diabetes mellitus, and repetitive strain injury. This could be due to the concern with comorbidity and the pursuit of a better quality of life. On the contrary, bad or very poor sleep quality was associated with higher smoking rates. Smoking is associated with the development of sleep disorders^23^ and an increased insomnia incidence^24^.

The results of the present study should be interpreted with caution, and the objective of the study was to estimate the current prevalence of smoking some years ago. And this scenario may have changed. Furthermore, the associations found may be the result of reverse causality, since, for example, a person who smoked all his/her life and was diagnosed with hypertension may probably stop smoking. In addition, the results of our study indicate that longitudinal studies are crucial to establishing the cause of some chronic diseases that are associated with a decrease and others with an increase in the prevalence of smoking in adults over 50 years of age. Another limitation concerns gathering information from individuals who decline to participate, making it challenging to compare them with participants and assess selection bias. However, ELSI-Brazil employed an inverse sampling design to address nonresponse bias without increasing the sample size.

It can be concluded that the prevalence of current smoking among older adults aged 50 years and older in Brazil was 17.04%. Factors associated with increased smoking prevalence in this age group included male gender, living without a partner, lower education level, the presence of chronic obstructive pulmonary disease, depression, poor quality of sleep, and alcohol intake. Participants’ conditions associated with the decrease in smoking prevalence were systemic arterial hypertension, hypercholesterolemia, and repetitive strain injury.
